# Illuminating the microbial ‘dark matter’ in obesity: integrated multi-omics approaches to characterize uncultured taxa and their potential metabolic crosstalk with host pathways

**DOI:** 10.3389/fmicb.2026.1940402

**Published:** 2026-10-20

**Authors:** Nan Zhang, Yuting Jin, Wenbo Ren, Jing Huang, Taiyu Zhai

**Affiliations:** Department of Clinical Laboratory, The First Hospital of Jilin University, Changchun, China

**Keywords:** gut microbiota, metabolic, microbial dark matter, multi-omics, obesity

## Abstract

**Background:**

Obesity is a global health challenge shaped by interactions among host genetics, diet, lifestyle, and gut microbial ecology. Although reference genome catalogues and cultivation approaches have expanded knowledge of the human gut microbiome, many intestinal microorganisms remain uncultured, poorly characterized, or functionally unresolved. These microorganisms are collectively referred to as microbial “dark matter,” a conceptual category rather than a defined taxonomic group encompassing diverse lineages, including members of Patescibacteriota, historically referred to collectively as the Candidate Phyla Radiation (CPR), with Saccharibacteria representing a historically used designation for a lineage within this broader group.

**Objective:**

This review examines the potential relevance of uncultured and poorly characterized gut microorganisms—collectively referred to here as microbial “dark matter”—to obesity-associated microbial ecology and metabolism. We focus on how integrated multi-omics approaches can identify candidate microbial functions, metabolic interactions, and host-associated pathways, while distinguishing genomic prediction and ecological association from demonstrated activity and causal effects. Particular attention is given to the evidence gaps surrounding microbial metabolite production or transformation by poorly characterized taxa and to experimental strategies required for functional and causal validation.

**Design:**

We summarize evidence on the ecological characteristics, genome reduction, metabolic dependencies, and potential host–microbe interactions of uncultured microorganisms. We discuss microbial metabolites, including short-chain fatty acids and bile acid derivatives, while distinguishing established microbiome–host mechanisms from functions specifically attributed to uncultured taxa. We also evaluate cultivation, gnotobiotic models, organoids, and microbial engineering for experimental validation.

**Results:**

Uncultured microorganisms represent an important but incompletely characterized component of the gut ecosystem. Genome-resolved and single-cell approaches have revealed substantial metabolic diversity and ecological dependencies. Some uncultured lineages may participate in carbohydrate metabolism, metabolite exchange, and bile acid transformation. However, direct evidence linking specific uncultured taxa to obesity phenotypes remains limited, with many proposed mechanisms based on genomic prediction, multi-omics associations, or extrapolation from better-characterized microorganisms. Their roles in antibiotic resistance and environmental-stressor responses also remain emerging rather than established mechanisms of obesity.

**Conclusion:**

Microbial dark matter represents an unresolved dimension of obesity-associated microbiome research. Integrated multi-omics can generate functional hypotheses, but causal attribution requires complementary experimental validation. A function-oriented framework distinguishing established evidence, mechanistic inference, and future hypotheses may provide a rigorous foundation for understanding uncultured components of the gut microbiome.

## Graphical Abstract

**Figure:**
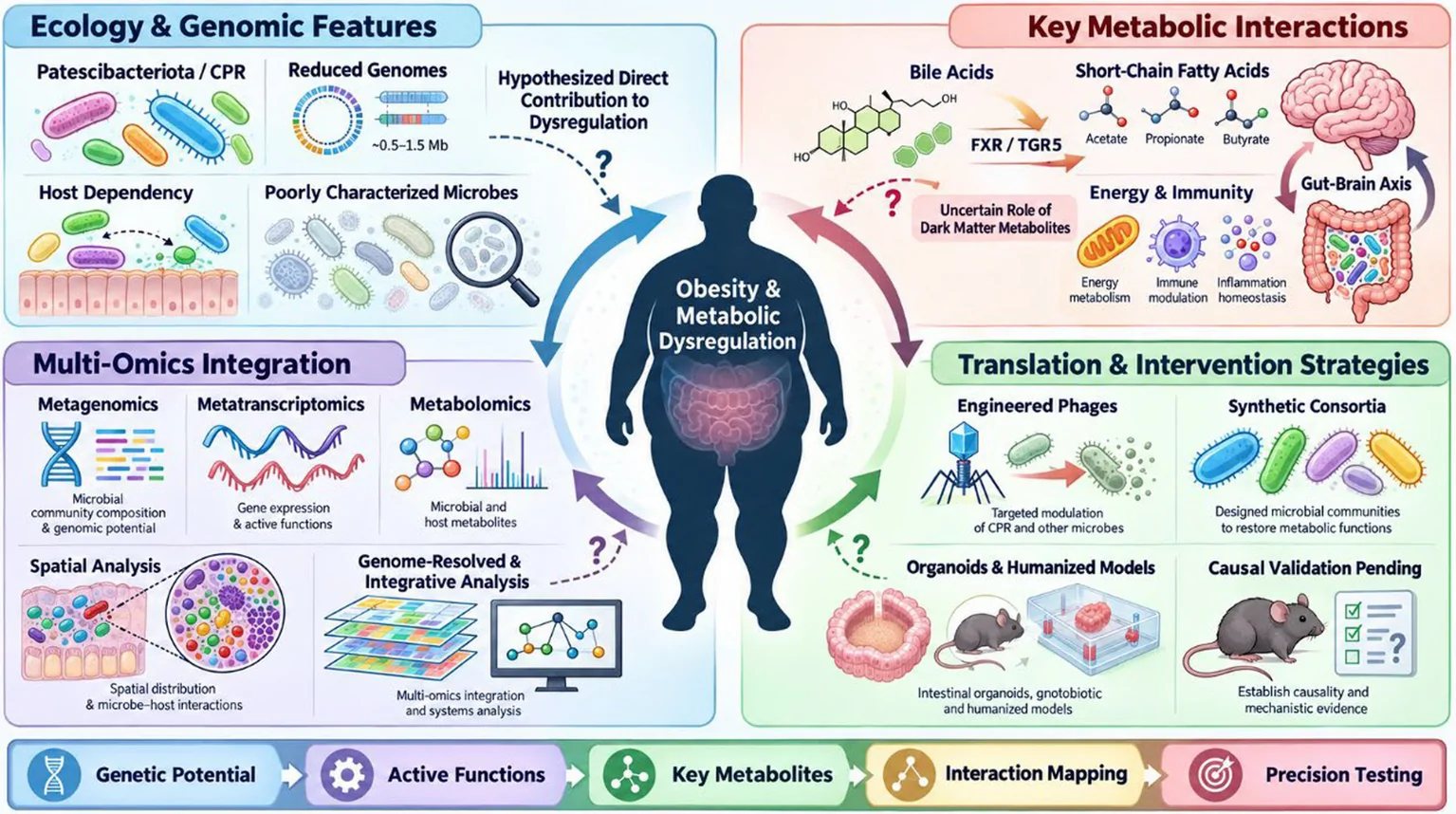
Decoding the potential role of microbial “dark matter” in obesity.

## 1 Introduction

Obesity is a multifactorial metabolic disorder shaped by interactions among genetic susceptibility, dietary exposure, host metabolism, lifestyle, and environmental factors. Increasing evidence indicates that the gut microbiome contributes to this complex network by influencing nutrient processing, immune homeostasis, metabolite production, and host signaling (Hall et al., 2017; Harley and Karp, 2012; Zhao, 2013). Although the gut microbiome has therefore become an important target of obesity research, its functional complexity extends beyond the taxa that can be readily cultured and experimentally characterized.

Early studies of the gut microbiome in obesity primarily examined differences in microbial community composition and the associations of relatively well-characterized taxa with metabolic phenotypes. Subsequent intervention studies have also investigated individual bacterial strains and probiotic approaches in experimental models of obesity and metabolic dysfunction (Pinart et al., 2021; Baek et al., 2023; Stojanov et al., 2020; Zheng et al., 2024). Although these studies have provided important insights into associations between gut microbial composition and host metabolic health, taxon-centered approaches capture only part of the functional complexity of the intestinal ecosystem. Microbial populations that remain difficult to culture or assign to well-characterized reference genomes may contain substantial functional diversity that is not captured by conventional approaches. The term “microbial dark matter” is useful in this context as a conceptual designation for such poorly characterized microbial diversity, rather than as a taxonomic entity.

Cultivation remains an important bottleneck because many gut microorganisms depend on specific physicochemical conditions, nutrients, metabolites, or interactions with neighboring organisms that are difficult to reproduce in isolation (Thomas et al., 2017; Jung et al., 2025). At the same time, the rapid expansion of reference genome collections and metagenomic sequencing has demonstrated that uncultured or poorly characterized organisms can be investigated without requiring immediate isolation in pure culture (Zou et al., 2019; Pasolli et al., 2019; Kim et al., 2022). These developments have shifted the central question from “How can these organisms be cultured?” toward “How can their functions be reliably attributed and experimentally tested?” This methodological transition is supported by complementary technologies. Advanced cultivation systems can recreate selected aspects of the intestinal environment and may enable the recovery of previously inaccessible organisms (Jung et al., 2025); Metagenomic and long-read sequencing, together with improved assembly and binning strategies, can recover genomes from uncultured populations and provide estimates of their metabolic potential (Zou et al., 2019; Pasolli et al., 2019; Kim et al., 2022). However, genomic potential should not be equated with demonstrated physiological activity. Determining whether predicted pathways are expressed and contribute to host-associated phenotypes requires integration with transcriptomic, proteomic, metabolomic, spatial, and experimental evidence.

The term “microbial dark matter” therefore describes an important knowledge gap rather than a predefined group of pathogenic or beneficial organisms. Uncultured populations may participate in nutrient exchange, cross-feeding, host-associated metabolism, or other ecological interactions, but the direction and magnitude of their effects on obesity cannot be inferred from unculturability alone. Some may contribute to disease-associated metabolic states, whereas others may represent adaptive or neutral components of the ecosystem. Current microbiome research increasingly combines 16S profiling, shotgun metagenomics, metatranscriptomics, metabolomics, single-cell sequencing, and spatial omics (Kumar et al., 2023). Each approach provides a different layer of information but also introduces specific limitations in taxonomic resolution, functional attribution, temporal interpretation, metabolite assignment, or spatial resolution (Rehner et al., 2025; Shen et al., 2025; Dell'olio et al., 2024). Their integration can therefore strengthen inference by linking microbial identity or genomic potential with transcriptional activity, metabolite profiles, ecological context, and host-associated localization. Importantly, however, multi-omics integration should be viewed as a framework for functional attribution and mechanistic hypothesis generation rather than as a stand-alone method for establishing causality.

Accordingly, the major challenge is not merely to expand the catalog of uncultured taxa, but to determine which populations encode, express, and causally mediate obesity-relevant functions. This review therefore focuses on how integrated multi-omics approaches can connect microbial genomes with metabolic activity, ecological interactions, spatial organization, and host responses. We distinguish established microbiome–host mechanisms from hypotheses specifically attributable to uncultured taxa and discuss the experimental strategies required to move from association to causal inference. By placing microbial dark matter within an evidence-based ecological and mechanistic framework, we aim to clarify both its potential contribution to obesity and the limitations that currently constrain translational application.

## 2 Emerging evidence for the potential metabolic functions of uncultured microorganisms

Conventional microbiological studies have preferentially characterized organisms that can be readily cultivated and experimentally manipulated. In contrast, genome-resolved metagenomics has revealed numerous phylogenetically diverse lineages that remain difficult to cultivate or incompletely characterized. Patescibacteriota lineages are prominent examples of such lineages and are notable for their highly streamlined genomes and extensive metabolic dependencies (Maatouk et al., 2022; Maatouk et al., 2023; Maatouk et al., 2021; Tian et al., 2020; Naud et al., 2022). Patescibacteriota lineages are often characterized by small, highly streamlined genomes and extensive metabolic dependencies, although genome size and functional repertoires vary among lineages (Maatouk et al., 2022; Maatouk et al., 2023; Maatouk et al., 2021; Tian et al., 2020). Some genomes retain genes encoding carbohydrate-active enzymes, suggesting that selected CPR populations may participate in carbohydrate utilization or metabolite exchange within microbial communities. However, the presence of these genes does not establish their expression or quantitative contribution to carbohydrate fermentation in the human gut. Studies from diverse ecosystems indicate that Patescibacteriota can occupy a broad range of ecological niches, including environments in which close associations with other microorganisms may be important for survival. These observations illustrate the ecological versatility and dependency patterns of the lineage, but environmental enrichment alone does not establish a role in human obesity. Genome-resolved analyses of Patescibacteriota have revealed extensive metabolic streamlining, including loss or incompleteness of central metabolic pathways and dependence on neighboring organisms for essential metabolites (Jonas et al., 2025). Such dependencies provide a mechanistic basis for cross-feeding and episymbiotic interactions. Some uncultured or poorly characterized lineages may possess metabolic functions relevant to bile acid transformation, but direct evidence for such activities in gut-associated CPR/Saccharibacteria remains limited.

Saccharibacteria, historically referred to as TM7, represents a well-studied lineage within the broader Patescibacteriota/CPR radiation and has been detected in mammalian-associated microbial communities. A study has revealed that Saccharibacter substantially enhances energy-metabolism efficiency through the acquisition of the arginine deiminase system (ADS). This system empowers it to efficiently utilize arginine for ATP production, thereby conferring a competitive edge in nutrient-rich mammalian niches (Tian et al., 2022). These findings indicate that the CPR lineage possesses a certain capacity for metabolic adaptation. In addition, advanced cultivation strategies have also enabled the enrichment of previously inaccessible microorganisms from challenging environments, illustrating the potential of cultivation innovation to complement genome-based approaches.

Some poorly characterized microbial lineages may encode functions relevant to bile acid transformation, but direct taxon-resolved evidence linking specific uncultured gut populations to bile acid modification remains limited (Hua et al., 2021; Marcy et al., 2007). Such activities could theoretically influence the pool of microbial bile acid derivatives available to interact with host receptors such as TGR5. Nevertheless, the contribution of these specific uncultured populations to bile acid-mediated metabolic phenotypes in obesity remains to be established. Multi-omics studies in individuals with obesity have also linked altered microbial diversity and tryptophan metabolism to host neuroendocrine signaling (Barone et al., 2022). Changes in microbial tryptophan metabolism may affect the availability of indole derivatives, serotonin-related metabolites, or kynurenine-pathway intermediates, although the contribution of specific uncultured taxa remains difficult to assign. Thus, the gut-brain axis represents a plausible area in which poorly characterized microbial populations could contribute to host metabolic regulation.

Collectively, current multi-omics evidence suggests that the uncultured taxa within microbial “dark matter” may represent an overlooked component in the energy and metabolic networks associated with obesity. Genomic and metabolic predictions suggest that these populations could potentially participate in energy harvesting, metabolite exchange, and host-associated metabolic signaling. However, these predictions require functional validation to determine whether specific uncultured populations actively contribute to obesity-associated metabolic phenotypes.

## 3 Multi-omics integration for functional attribution and causal hypothesis generation

While single-omics approaches provide valuable insights, they face intrinsic limitations in comprehensively capturing the functional dynamics of complex intestinal ecosystems, particularly regarding uncultured microbial populations. Although integrating multi-omics strategies is imperative for generating robust mechanistic hypotheses and strengthening functional inferences, it is essential to acknowledge that multi-omics platforms primarily reveal associative networks. To definitively transition from these correlations to causal linkages between uncultured microbial functions and host obesity phenotypes, systems-level omics analyses must be complemented by downstream experimental platforms, such as gnotobiotic animal models, organoid validation, or targeted microbial engineering.

For instance, while 16S rRNA gene sequencing is widely applied for taxonomic profiling, it lacks the resolution to elucidate specific functional genes or metabolic pathways. Consequently, this approach merely identifies the presence of uncultured taxa without revealing their metabolic capabilities or ecological roles. Although metagenomics addresses this functional gap, the immense complexity of the gut ecosystem presents significant bioinformatic challenges. During metagenomic assembly, sequences from low-abundance taxa or highly similar strain variants are frequently misassembled or binned incorrectly. This limitation disproportionately affects microorganisms with ultra-small genomes, such as the CPR, which is currently classified under the superphylum Patescibacteriota. The unique genomic architectures of these organisms—often characterized by severe genome reduction, atypical ribosomal structures, and distinct codon usage—can be inadequately captured by standard bioinformatics pipelines. As a result, critical segments of their genetic repertoire may be fragmented or lost during assembly, leading to incomplete or biased functional annotations (Kutuzova et al., 2024).

To improve genome recovery from complex metagenomic datasets, reconstruction of metagenome-assembled genomes (MAGs) has become an important genome-resolved approach. MAGs are genome bins reconstructed from metagenomic sequence data through assembly, binning, refinement, and quality assessment (Chen Z. et al., 2024; Benoit et al., 2024; Zhou et al., 2022). By grouping contigs inferred to originate from the same microbial population, this workflow can recover partial to near-complete genomic representations of microorganisms that remain difficult to cultivate. These reconstructed genomes can be used to infer encoded metabolic potential; however, predicted genes or pathways do not by themselves establish physiological activity (Zhou et al., 2022).

To dynamically elucidate how these genetic potentials are translated into active functions, integrating metatranscriptomics and metabolomics is imperative. Metatranscriptomics enables the profiling of actively transcribed genes within microbial communities under metabolic states such as obesity. Conversely, metabolomics allows the quantitative detection of intestinal end-metabolites. By performing integrative correlation analyses between these layers-for instance, linking transcriptionally active gene clusters (such as polysaccharide utilization loci, PULs) with corresponding alterations in specific gut metabolites-researchers can comprehensively evaluate microbial functional dynamics. This multi-omics correlation shifts the analytical focus from static genomic predictions to dynamic assessments of functional profiles executed under specific physiological conditions, thereby enabling more reliable inferences regarding their biological roles. Furthermore, MAG-informed metatranscriptomic profiling empowers the assessment of functional states across specific populations, including uncultured microorganisms (Yan et al., 2024). Cross-omics studies in obesity-related and metabolic contexts have demonstrated that integrated transcriptomic, microbiome, and metabolomic analyses can reveal coordinated molecular and metabolic changes across biological layers. Such studies are useful for identifying candidate pathways and generating mechanistic hypotheses; however, they do not by themselves establish that specific metabolites are produced by defined uncultured microbial populations. Therefore, these associations should be considered hypothesis-generating evidence that requires taxon-resolved and experimental validation (Liao et al., 2023; Pilger et al., 2024; Sobolev et al., 2019).

Furthermore, spatial metabolomic imaging technologies offer a useful approach for mapping host-microbe metabolic crosstalk. By leveraging these platforms, researchers can directly visualize the precise spatial localization of microbial metabolites-including specific lipids and amino acids-within intestinal crypts, lymphoid follicles, and adipose tissue, all without disrupting native tissue architecture. Consequently, this provides intuitive insights into potential microenvironmental interaction sites between microbial metabolites and host cells (Vandereyken et al., 2023). To rigorously determine whether these spatially resolved molecules directly drive host metabolic modifications, complementing spatial profiling with *in vitro* experimental platforms, such as organoid co-culture systems, is essential for causal validation. This integrative strategy not only elucidates the precise functional impacts of these metabolites on host cellular responses but also facilitates the transition from spatial correlations to robust, mechanism-based evidence (Legnini et al., 2023).

In conclusion, multi-omics integration serves as a cornerstone strategy for systematically elucidating the functional roles of microbial “dark matter” in the context of obesity (Coleman et al., 2025). By integrating metagenomics, metatranscriptomics, metabolomics, and spatial profiling, researchers can obtain complementary evidence concerning encoded functional potential, transcriptional activity, metabolite abundance, and spatial context. Concordance across these analytical layers can strengthen functional hypotheses, but it does not establish a linear causal chain from gene presence to metabolite production or host phenotype (Zhao et al., 2022; Chen S. et al., 2024; Lundgren et al., 2023). Metabolite abundance does not by itself identify the producing organism or directly measure metabolic flux. Robust causal attribution therefore requires complementary experimental validation. Approaches such as stable-isotope tracing, strain-resolved genomic linkage, controlled perturbation, and direct measurement of host responses are required to establish producer identity, metabolic flux, and host consequence.

## 4 Ecological and genetic attributes of the obesity-associated “microbial dark matter”

The integrated application of multi-omics technologies can substantially expand our understanding of gut microbial diversity and help characterize the genomic and metabolic features of uncultured or poorly characterized microorganisms associated with obesity. Rather than being considered merely as uncharacterized background components of the gut ecosystem, these microorganisms may contribute to the metabolic milieu of the gut through genome streamlining, niche adaptation, metabolic dependencies, and interactions with neighboring microbial populations. However, genomic and ecological features alone do not establish their functional activity or causal involvement in obesity. Therefore, integrating their phylogenetic distribution, genomic characteristics, expressed functions, metabolic dependencies, and spatially resolved activities provides an important framework for investigating their potential roles in obesity-associated microbial ecology and for addressing the broader “microbial dark matter dilemma.”A central objective of this approach is to move beyond the detection of uncultured taxa toward identifying which populations are metabolically active, which functions they express, and whether those functions causally influence host metabolic phenotypes. This distinction will be essential for translating multi-omics observations into experimentally testable mechanisms.

### 4.1 Ecological niche traits, phylogenetic distribution, and potential associations with obesity

Metagenomic investigations have identified enrichment patterns of certain difficult-to-cultivate or poorly characterized microbial groups in the gut microbiota of individuals with obesity, suggesting potential ecological associations between these populations and obesity-related metabolic states (Li et al., 2023; Shahab and Shahab, 2022). Among these lineages, members of Patescibacteriota, historically referred to as the CPR and including the lineage historically known as Saccharibacteria, represent prominent examples of uncultured or poorly characterized microbial diversity. Large-scale human microbiome studies have identified associations between BMI and specific SCFA-producing taxa, although the direction and magnitude of these associations vary across taxa and populations (Zahavi et al., 2023; Ecklu-Mensah et al., 2023). This finding indicates an association between gut microbial metabolic capacity and host adiposity, but it should not be interpreted as evidence that CPR proliferation directly sustains obesity. Moreover, the relationship between individual microbial taxa, SCFA production, and obesity-related phenotypes is likely to depend on microbial community composition, dietary substrates, and host metabolic context. CPR bacteria are characterized by highly streamlined genomes and small cell sizes and frequently exhibit metabolic dependencies on neighboring microorganisms. Some CPR members can attach to the surfaces of larger and more metabolically autonomous microorganisms and obtain essential nutrients, including amino acids and nucleotides, through these associations (Maatouk et al., 2022; Mclean et al., 2020). Such ecological dependencies may facilitate metabolite exchange and cross-feeding within microbial communities. In obesity-associated metabolic environments, altered nutrient availability and host dietary patterns may influence the ecological distribution of metabolically dependent microorganisms. These conditions could potentially affect the abundance or activity of CPR and other uncultured populations with specialized substrate-utilization capabilities. However, whether an increase in these populations enhances overall microbial metabolic flux, intestinal energy harvest, or positive energy balance remains to be experimentally established (Kellow et al., 2014). Importantly, the ecological enrichment of uncultured taxa should be distinguished from functional and causal evidence. Future studies integrating strain-resolved metagenomics, metatranscriptomics, metabolomics, and spatial profiling with targeted perturbation will be required to determine whether specific uncultured populations actively contribute to obesity-associated metabolic phenotypes.

Conversely, studies comparing lean and obese populations have reported differences in the relative abundance of multiple microbial taxa and community configurations (Crovesy et al., 2020; Amabebe et al., 2020; Renall et al., 2023). However, the identity and consistency of lean-associated poorly characterized lineages vary among cohorts, and current evidence is insufficient to conclude that specific uncultured lineages directly maintain intestinal barrier integrity or promote metabolic health. These observations should therefore be interpreted as ecological associations requiring taxon-resolved functional validation (Fumagalli et al., 2025). Overall, the gut microbiota of individuals with obesity is frequently characterized by the overgrowth of Firmicutes and Proteobacteria, which are implicated in heightened energy absorption and exacerbated low-grade inflammation (Palmas et al., 2021; Tu et al., 2025; Sugama et al., 2021). The relative enrichment of specific uncultured microbial lineages in lean individuals potentially suggests a microbiota composition that is more favorable for metabolic homeostasis and immune equilibrium (Stenman et al., 2016). The relative enrichment of specific poorly characterized or uncultured lineages in lean individuals may therefore reflect ecological configurations associated with metabolic and immune homeostasis, although the consistency and functional significance of these associations remain to be determined. This differential distribution implies that distinct components within the gut microbiota’s “dark matter” may participate in antagonistic ecological processes that facilitate energy storage and uphold metabolic health. Taken together, these differential distributions support the hypothesis that poorly characterized components of the gut microbiota may occupy distinct ecological niches in lean and obese states and could contribute to differences in metabolite exchange, host–microbe interactions, and metabolic phenotypes. However, enrichment or depletion alone does not demonstrate functional activity or causality. Future efforts need to integrate strain-resolved metagenomics with metatranscriptomics, metabolomics, and targeted experimental interventions to determine whether specific uncultured microorganisms actively contribute to obesity-related metabolic dysfunction.

### 4.2 Genetic underpinnings of genome reduction and metabolic dependency

The pronounced tendency toward genome reduction and metabolic streamlining is a characteristic feature of many members of the uncultured or poorly characterized microbial fraction, particularly within Patescibacteriota, and is closely associated with their distinctive ecological strategies. Genome sizes in these lineages can be substantially smaller than those of many free-living bacteria, and their metabolic repertoires are often highly streamlined, with some genomes lacking complete pathways for the autonomous synthesis of essential metabolites, including amino acids, lipids, and nucleotides (Chiriac et al., 2022; He et al., 2025; Castelle et al., 2018). Such genome reduction is generally interpreted as the outcome of long-term evolutionary adaptation to specific ecological niches and recurrent dependence on metabolites or cellular functions supplied by other community members; however, its precise evolutionary drivers may vary among lineages and environments. The resulting metabolic dependencies can favor close associations with neighboring microorganisms and, in some cases, obligate episymbiotic or host-associated lifestyles. Genomic analyses support such metabolic interdependencies across multiple Patescibacteriota lineages, although much of the available evidence derives from genome-resolved studies across diverse environments rather than obesity-specific human gut cohorts (Chiriac et al., 2022; Castelle et al., 2018).

Importantly, genome reduction in many Patescibacteriota lineages extends into central metabolic functions. For example, many CPR bacteria lack complete sets of genes for the tricarboxylic acid (TCA) cycle and have reduced or incomplete biosynthetic pathways for several essential amino acids, vitamins, and coenzymes (Maatouk et al., 2023; Castelle et al., 2018). These losses are generally interpreted as consequences of evolutionary streamlining and adaptation to ecological niches in which metabolites or cellular functions can be obtained from neighboring organisms, although the extent and nature of such dependencies vary across Patescibacteriota lineages. Genome streamlining may be accompanied by retention or enrichment of genes involved in functions that are advantageous within particular ecological contexts, including carbohydrate-active enzymes in some lineages (Castelle et al., 2018). Such functional specialization could facilitate niche partitioning, substrate utilization, and metabolic exchange with neighboring microorganisms, but the presence of these genes alone does not establish their expression or ecological importance in the human gut. Thus, genome reduction provides a genomic basis for metabolic dependency and close ecological interactions in some Patescibacteriota lineages. These features raise the possibility that CPR-associated populations may function as metabolically interconnected components of the gut microbial community, but whether they constitute active and functionally important metabolic modules in obesity-associated ecosystems requires direct validation.

### 4.3 Integrating ecological and genetic traits to define potential functions

Uncultured and poorly characterized microorganisms do not constitute a biologically uniform group. Some Patescibacteriota lineages exhibit genome reduction, metabolic dependency, and episymbiotic lifestyles, whereas other uncultured taxa may have distinct ecological strategies. In obesity-associated environments, dietary and host-derived changes could alter the distribution or activity of metabolically dependent microorganisms, but whether these ecological changes increase microbial energy harvest or contribute to host energy balance remains uncertain. Integrating genomic, transcriptional, metabolomic, and spatial evidence with targeted perturbation will be necessary to determine whether specific uncultured populations exert measurable effects on host metabolic phenotypes (Castelle et al., 2018). Conversely, uncultured or poorly characterized microbial lineages enriched in lean individuals may occupy ecological niches associated with host-derived substrates or microbial cross-feeding networks. Their enrichment may correlate with barrier function and immune-metabolic homeostasis, but direct evidence that these taxa themselves maintain barrier integrity or actively promote metabolic health remains limited. Thus, integrating genomic traits with ecological context may help identify candidate dark-matter populations and functions, while experimental perturbation is required to determine whether these associations reflect active contributions to host metabolic phenotypes. Future research should analyze these microorganisms within ecological and metabolic networks involving the host, cultivable bacteria, and other uncultured microorganisms, rather than treating them as independent functional entities. Such an approach will help determine whether specific populations represent potential contributors, adaptive consequences, or regulatory components of obesity-associated microbial states.

## 5 Potential mechanisms of dark matter-host metabolic interactions

Although gut microbiota “dark matter” presents substantial challenges for cultivation and functional characterization, the available genomic and metagenomic evidence indicates that at least some uncultured or poorly characterized microorganisms possess metabolic capacities that could influence microbial community functions. Their encoded enzymes and metabolic pathways may contribute to the production, transformation, or consumption of microbial metabolites, potentially linking uncultured populations to broader microbial metabolic networks and host-associated signaling pathways. Microbial metabolites, including short-chain fatty acids, bile acid derivatives, tryptophan metabolites, and other small molecules, are important mediators of microbiota–host communication and can influence host metabolic, immune, and neuroendocrine processes (Dinglasan et al., 2025; Wahlström et al., 2016; Furusawa et al., 2013; Lai et al., 2021). However, these established microbiota–host pathways should not be attributed specifically to microbial “dark matter” unless the responsible uncultured taxa or functions have been directly identified. A major challenge is therefore to determine which uncultured microorganisms actively produce or modify host-relevant metabolites, whether these functions are expressed *in vivo*, and whether perturbation of the responsible taxa or metabolic pathways alters obesity-associated phenotypes. Integrating metagenomics, metatranscriptomics, metabolomics, spatial profiling, and targeted experimental perturbation may help establish mechanistic links, while causal inference ultimately requires validation in appropriate experimental models.

### 5.1 Bile acid metabolism as a potential Interface between microbial activity and host signaling

Bile acid transformation provides a plausible interface through which poorly characterized gut microorganisms could interact with host metabolic signaling. However, direct evidence specifically linking uncultured taxa to bile acid transformation remains limited. Current evidence is largely based on genomic or metagenomic observations suggesting that some poorly characterized populations may encode functions potentially involved in bile acid modification, whereas taxon-resolved evidence demonstrating *in vivo* expression, substrate conversion, or quantitative contribution to the intestinal bile acid pool remains scarce.

At the host level, established bile acid pathways involving FXR and TGR5 provide a biological framework for investigating these potential interactions. Alterations in secondary bile acid composition have been associated with obesity-related metabolic phenotypes, but these associations are influenced by microbial community composition, diet, and host metabolic state (Wahlström et al., 2016; Wei et al., 2020; Jung et al., 2022; Zhao et al., 2023). These established bile acid–host pathways provide a biologically plausible framework for investigating whether uncultured microbial populations contribute to host metabolic phenotypes through bile acid transformation. Nevertheless, such pathways should not be attributed specifically to microbial “dark matter” unless the responsible taxa or microbial functions have been directly identified.

The physiological relevance of bile acid–microbiota interactions has been investigated in diverse metabolic disorders. In obesity and metabolic syndrome, alterations in the composition of the bile acid pool, including changes in the relative abundance of non-12-hydroxy bile acids, have been associated with metabolic phenotypes (Wei et al., 2020). Taken together, bile acid metabolism represents a plausible interface through which microbial community activity may influence host metabolic and immune signaling. A key unresolved question is whether specific uncultured taxa make measurable contributions to this bile acid pool and whether perturbing these taxa or their bile acid–modifying functions alters obesity-associated phenotypes (Figure 1).

**Figure 1:**
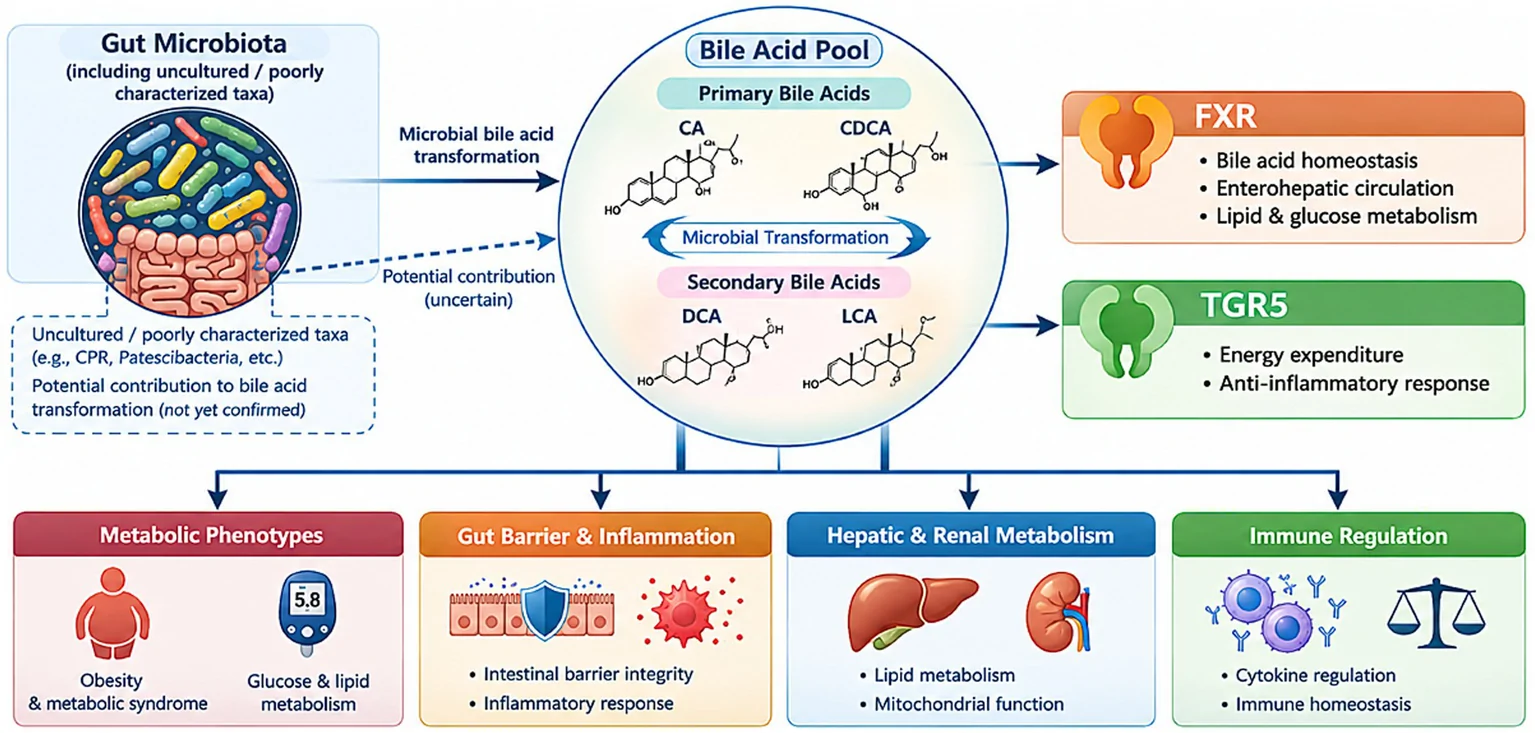
Potential contribution of uncultured gut microorganisms to microbiota-mediated bile acid metabolism and host signaling. Gut microbial communities transform primary bile acids into secondary bile acids, which can modulate host signaling through FXR and TGR5 and thereby influence metabolic, immune, intestinal barrier, and hepatic processes. Uncultured and poorly characterized taxa, including members of the Patescibacteriota lineages, may contribute to bile acid transformation through predicted or as-yet-uncharacterized metabolic functions; however, their *in vivo* activity and causal contribution to obesity-associated phenotypes remain uncertain and require experimental validation. Solid arrows indicate established gut microbiota–bile acid pathways, whereas dashed arrows indicate potential or uncertain contributions of uncultured taxa.

### 5.2 Short-chain fatty acids as important mediators of immune–metabolic homeostasis

Short-chain fatty acids (SCFAs), particularly acetate, propionate, and butyrate, are major products of microbial carbohydrate fermentation and important mediators of gut–host metabolic and immune communication. Their established effects include regulation of intestinal barrier function, immune responses, and host energy metabolism through mechanisms involving G-protein-coupled receptors and epigenetic pathways (Mukhopadhya and Louis, 2025). However, the relevance of these established SCFA effects to microbial “dark matter” depends on whether specific uncultured or poorly characterized microorganisms measurably contribute to SCFA production, consumption, or cross-feeding.

Current evidence suggests that some poorly characterized microbial lineages may encode carbohydrate-degradation or fermentation-associated functions, providing a genomic basis for their potential participation in SCFA-related metabolic networks. Nevertheless, the presence of carbohydrate-active enzymes or fermentation-related genes does not establish transcriptional activity, metabolite production, or a quantitative contribution to the community SCFA pool. In particular, direct human-gut evidence linking a defined uncultured lineage to a measurable fraction of acetate, propionate, or butyrate production remains limited (Furusawa et al., 2013; Arpaia et al., 2013). Simultaneously, SCFAs can modulate pro-inflammatory signaling pathways, including nuclear factor kappa B (NF-κB), through interactions with G-protein-coupled receptors (GPCRs), including GPR43 and GPR41, which are expressed by intestinal epithelial and immune cells. Such receptor-mediated signaling may contribute to the regulation of inflammatory responses (Ganesan et al., 2022; Quinn et al., 2021; Lee et al., 2024; Mann et al., 2024).

Indirect evidence may therefore be interpreted at several levels. Genome-resolved metagenomics can identify candidate metabolic pathways; metatranscriptomics can determine whether these pathways are transcriptionally active; metabolomics can establish changes in the corresponding metabolite pools; and isotope-tracing or flux-based approaches may help determine whether a candidate lineage contributes directly to metabolite production. Spatially resolved approaches may further clarify whether candidate microorganisms and SCFA-associated metabolic activity occur within the same intestinal microhabitats.

Thus, the key unresolved question is not whether SCFAs influence host physiology, which is well established, but whether specific members of microbial dark matter make a measurable and biologically meaningful contribution to SCFA metabolism in the human gut, particularly in obesity. Addressing this question will require integration of genome-resolved multi-omics with metabolite flux measurements, stable-isotope tracing, spatial profiling, and targeted ecological or experimental perturbation. Until such evidence becomes available, potential SCFA contributions from uncultured microorganisms should be considered hypotheses rather than established mechanisms (Figure 2).

**Figure 2:**
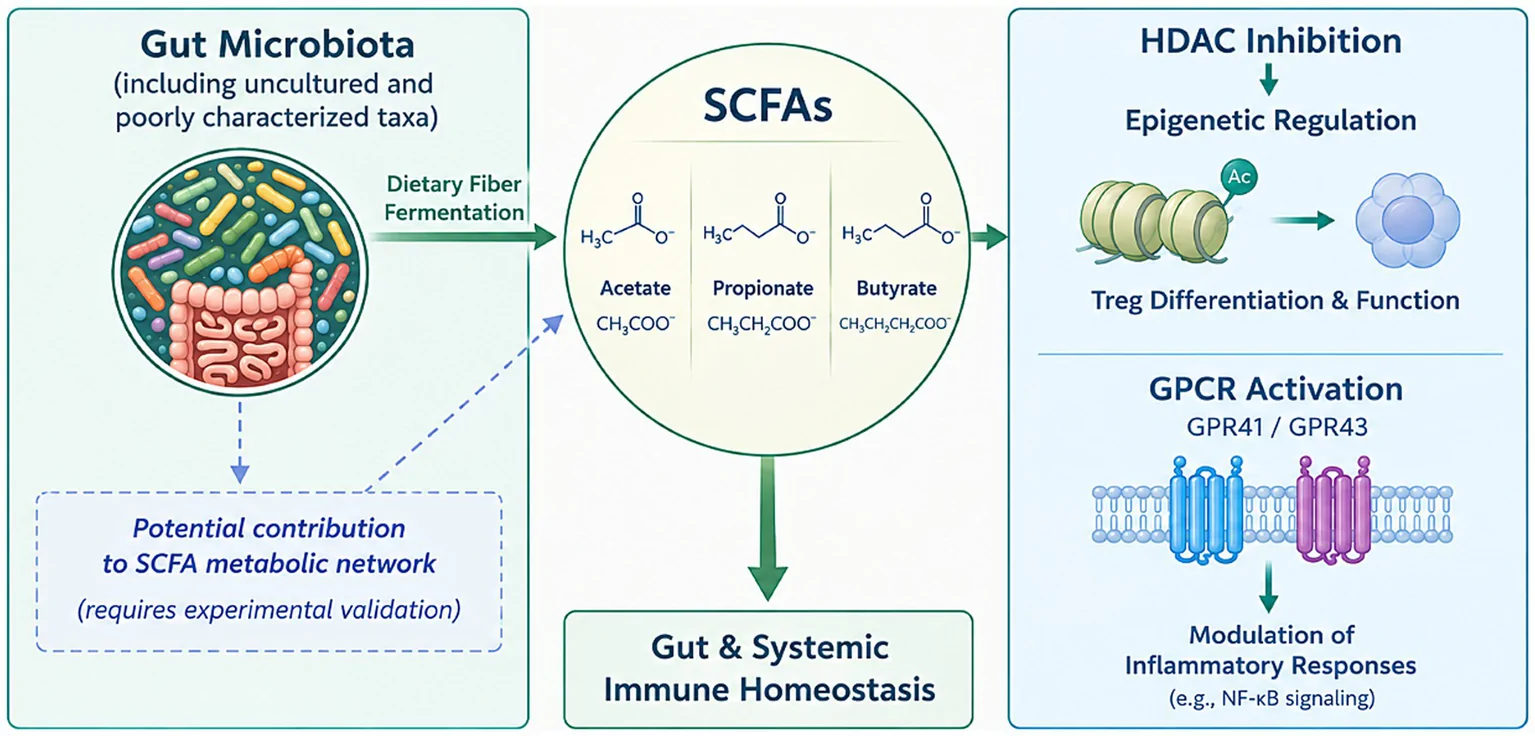
Established and potential links between gut microbial SCFA metabolism and immune-metabolic regulation. Dietary fiber is fermented by the gut microbiota to produce SCFAs, predominantly acetate, propionate, and butyrate. These metabolites can regulate host immune responses through mechanisms involving HDAC inhibition, epigenetic regulation, Treg differentiation, and activation of SCFA-responsive GPCRs such as GPR41 and GPR43. Uncultured and poorly characterized microorganisms may contribute to SCFA production, utilization, or cross-feeding within the gut microbial community; however, their specific quantitative and causal contributions to SCFA-mediated host effects remain uncertain. Solid arrows indicate established biological relationships, whereas dashed arrows indicate potential contributions requiring experimental validation.

### 5.3 Neuroactive signals and systemic metabolites as potential mediators of gut–organ communication

The metabolic activities of the gut microbiota can influence host physiology beyond the local intestinal environment through microbial metabolites and microbe–host signaling pathways. Microbial metabolites that enter the circulation may interact with distant organs, providing a plausible route through which gut microbial activity could contribute to systemic metabolic, immune, and neurophysiological regulation.

Neuroactive and circulating microbial metabolites provide another potential route through which poorly characterized gut microorganisms could influence host physiology. However, evidence specifically attributing neuroactive or systemic metabolite production to microbial “dark matter” remains limited. Current studies demonstrate that gut microbial communities can alter circulating and tissue-associated metabolites involved in gut–brain and systemic signaling, but these observations generally remain at the community level and do not establish the contribution of specific uncultured taxa (Lai et al., 2021; Toledo et al., 2025; Gophna et al., 2017; Kircher et al., 2022; Strandwitz et al., 2019).

Similarly, the microbial conversion of dietary choline or carnitine to trimethylamine (TMA), followed by host conversion of TMA to trimethylamine N-oxide (TMAO), represents an established microbiota–host metabolic pathway. However, current evidence does not establish that poorly characterized taxa are major contributors to TMA production or that their activity accounts for obesity-associated TMAO phenotypes (Lu et al., 2023; Dalla Via et al., 2019). Thus, established gut–brain, TMA/TMAO, and related systemic signaling pathways should be considered biological contexts in which dark-matter involvement can be investigated rather than evidence of a dark-matter-mediated mechanism.

In conclusion, microbial metabolites and microbe–host signaling pathways provide plausible mechanisms through which the gut microbiota can influence host physiology at both local and systemic sites, including the intestine, liver, adipose tissue, brain, and cardiovascular system. Bile acids, SCFAs, neuroactive metabolites, and TMAO represent important classes of microbiota-associated molecules with established or emerging roles in host signaling. Nevertheless, their production or modification should not be automatically attributed to microbial dark matter in the absence of taxon-resolved functional evidence. Future research should therefore focus not only on discovering previously uncharacterized metabolites but also on determining which uncultured populations produce, transform, or consume these molecules and how their activities interact with established microbial pathways during obesity development and progression. A central objective is to connect genomic potential with active microbial functions and experimentally validated host effects, thereby distinguishing ecological association from functional and causal contributions. Such an evidence-based framework may ultimately identify specific microbial populations, metabolic functions, or microbial–host interactions that can be targeted to modulate obesity and its associated complications (Figure 3).

**Figure 3:**
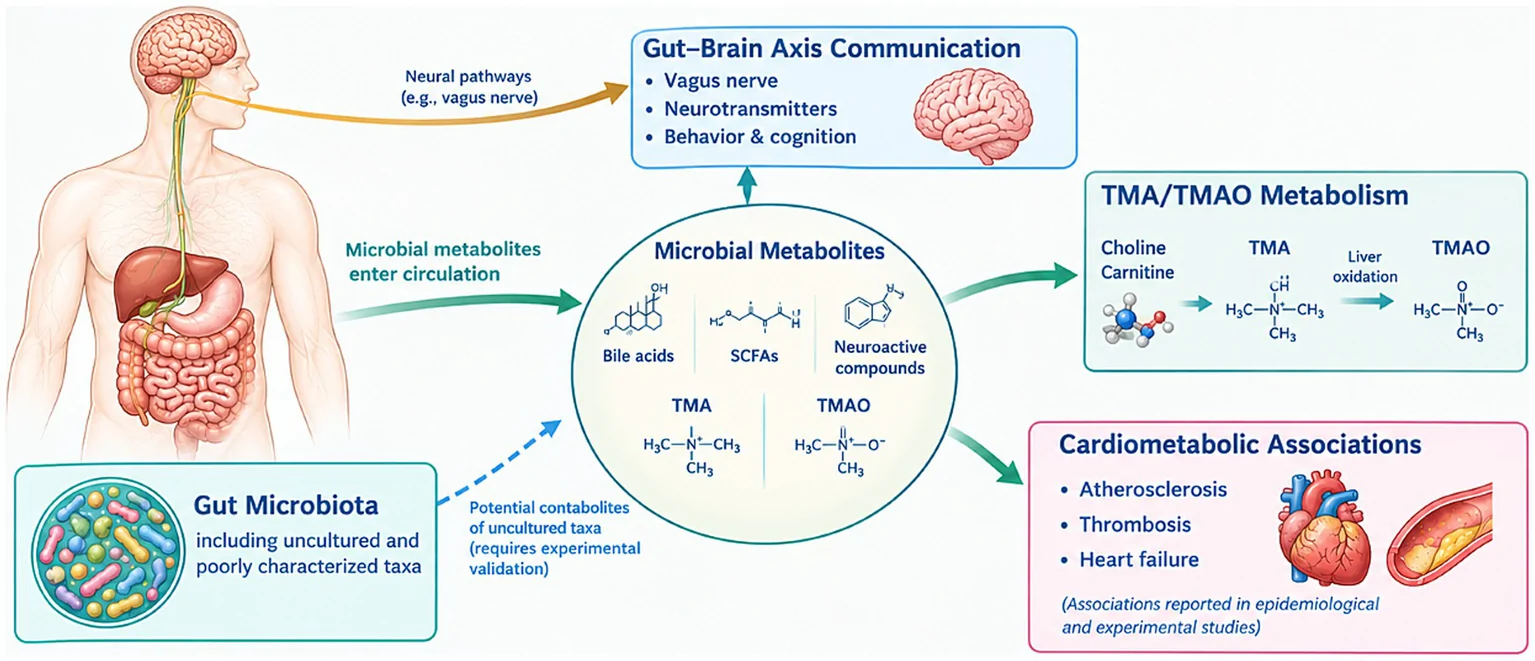
Potential contribution of uncultured gut microorganisms to systemic metabolite-mediated gut–organ communication. Gut microbial metabolism generates a diverse range of metabolites that can participate in communication between the intestine and distant organs through neural and systemic pathways. Microbial metabolites may influence the gut–brain axis through mechanisms involving the vagus nerve and neuroactive signaling, while metabolites such as trimethylamine (TMA) can be converted to trimethylamine N-oxide (TMAO) in the host liver and have been associated with cardiometabolic phenotypes. Uncultured and poorly characterized microorganisms may contribute to these metabolic networks through previously uncharacterized metabolic functions; however, their specific contributions to individual metabolite pathways and their causal effects on obesity-related phenotypes remain uncertain. Solid arrows indicate established or well-supported microbiota–host pathways, whereas dashed arrows indicate potential contributions of uncultured or poorly characterized taxa that require experimental validation.

## 6 Translational opportunities and technological challenges

The investigation of microbial “dark matter” within the gut microbiota may provide new opportunities for understanding and ultimately manipulating microbiota-associated functions relevant to obesity. However, its translation into therapeutic applications remains an emerging and largely exploratory field. Rather than assuming that uncultured microorganisms themselves can immediately serve as therapeutic targets, an important objective is to identify specific microbial functions that are reproducibly associated with obesity, are active *in vivo*, and can be experimentally manipulated. Many gut microorganisms remain difficult to isolate and cultivate using conventional techniques, limiting direct experimental characterization of their physiology and host interactions. This challenge supports a shift from a predominantly taxon-centered approach toward a function-oriented framework that integrates microbial genomic potential, expressed metabolic activities, ecological interactions, and host phenotypes. Such an approach may enable the development of interventions that target validated microbial functions without necessarily requiring cultivation of the corresponding microorganisms, while also highlighting the technological, biological, and safety barriers that must be addressed before clinical translation.

Given the difficulty of obtaining pure cultures for many gut microorganisms, function-oriented interventions represent a potential strategy for circumventing the cultivation bottleneck. The central concept is to reconstruct, substitute, or modulate experimentally validated microbial functions rather than requiring direct recovery of the corresponding microbial taxa. This approach shifts the research focus from a predominantly “strain-centered” framework toward a “function-centered” framework. However, such functional replacement should currently be considered a prospective research strategy rather than an established therapeutic approach for obesity, because the specific functions contributed by most uncultured taxa and their causal relevance to host phenotypes remain insufficiently defined. For disease-associated microbial populations whose functional contributions have been experimentally established, engineered bacteriophages or other targeted antimicrobial systems may provide a means of selectively reducing or inhibiting specific bacterial populations while minimizing disruption of surrounding microbial communities (Gao et al., 2018; Fujimoto et al., 2020). These approaches are particularly relevant to precision microbiome manipulation, but their applicability to uncultured obesity-associated taxa remains to be demonstrated *in vivo*. CRISPR-based antimicrobial systems, including RNA-targeting Cas13 approaches, have demonstrated the feasibility of sequence-specific targeting of bacterial transcripts and may produce selective antibacterial effects (Kiga et al., 2020). Such systems may target transcripts associated with antimicrobial resistance, although their applicability to uncultured obesity-associated taxa remains to be established.

Synthetic biology provides a complementary strategy for functional reconstruction by engineering microbial strains or defined microbial consortia to perform selected metabolic activities. Engineered microbial systems have been explored as programmable platforms for sustained production or delivery of therapeutic molecules and for modulation of disease-associated biological processes (Xie et al., 2025; Jin et al., 2025). In principle, such systems could be designed to reproduce selected functions hypothesized to be performed by uncultured microorganisms, including the production of specific metabolites or the transformation of potentially harmful compounds. However, this concept remains largely hypothetical in the context of obesity and requires validation through functional assays, controlled microbial perturbation, and physiologically relevant host models. Importantly, successful functional reconstruction should be evaluated not only by the restoration of an individual metabolic activity but also by its effects on community structure, metabolite networks, host physiology, and ecological stability.

However, the practical realization of function-oriented interventions remains constrained by substantial methodological and translational challenges. A critical priority is the high-resolution characterization of the functional properties of uncultured and poorly characterized microorganisms within the gut microbiome. Advances in microfluidic single-cell sorting and sequencing have enabled the recovery of genomic information from individual microbial cells, including previously inaccessible uncultured populations, thereby expanding our understanding of their genetic potential (Marcy et al., 2007). However, genomic potential alone does not establish whether predicted functions are expressed *in vivo*, nor does it demonstrate that these functions contribute causally to obesity-related phenotypes. Therefore, functional characterization should integrate population- or strain-resolved metagenomics with metatranscriptomics, proteomics, metabolomics, spatial profiling, and targeted perturbation approaches to distinguish genomic capacity from active microbial functions. Physiologically relevant experimental models, including intestinal organoids, organ-on-a-chip systems, and appropriately designed humanized animal models, may provide complementary platforms for investigating microbial–host interactions and evaluating candidate interventions. Although models incorporating human microbiota and/or human immune components may improve the assessment of human-specific interactions, their ability to reproduce the complexity of human obesity remains limited and requires careful validation. Thus, such models should be regarded as complementary preclinical tools rather than indispensable systems for establishing therapeutic efficacy. Moreover, bioengineering-based interventions face important biosafety and ecological challenges during translation. Engineered bacteriophages, for example, require systematic assessment of host range, off-target effects, horizontal gene transfer, resistance development, persistence, and potential disruption of the surrounding microbial ecosystem. The development of conditionally active or otherwise controllable phage systems may provide one potential strategy for restricting therapeutic activity to defined biological or environmental contexts. Such approaches could improve spatial and temporal control of microbial manipulation, but their feasibility, stability, specificity, and safety must be established through rigorous preclinical studies before clinical application.

In conclusion, the “microbial dark matter dilemma” in obesity research extends beyond the discovery of previously uncharacterized microorganisms and requires a systematic transition from detection to functional characterization, causal validation, and ultimately intervention. A multidimensional framework integrating iterative multi-omics, computational analyses, spatial approaches, and experimental perturbation can progressively refine functional maps of uncultured microbial populations. Organoids and organ-on-a-chip platforms can provide controlled physiological contexts for testing candidate microbial–host mechanisms, while animal models can complement these studies by assessing systemic metabolic effects and therapeutic responses. Synthetic biology and targeted delivery technologies may eventually enable precise manipulation of experimentally validated microbial functions; however, these approaches should currently be considered prospective strategies rather than established therapies for obesity. The central translational objective is therefore not simply to discover or eliminate microbial “dark matter,” but to identify specific uncultured populations or functions that are reproducibly associated with obesity, demonstrably active *in vivo*, causally linked to host phenotypes, and safely amenable to intervention. Establishing this evidence chain from genomic potential to active function and causal host effects will be essential for determining whether microbial “dark matter” can ultimately provide reliable therapeutic targets for obesity and its associated complications.
